# Mass-forming ischemic colitis mimicking colon cancer: A case report of early repeat colonoscopy

**DOI:** 10.1097/MD.0000000000050814

**Published:** 2026-09-25

**Authors:** Hyeon Seok Baek, Seung Young Seo

**Affiliations:** a Department of Internal Medicine, Jeonbuk National University Medical School, Research Institute of Clinical Medicine of Jeonbuk National University-Biomedical Research Institute of Jeonbuk National University Hospital, Jeonju, Jeollabuk-do, South Korea.

**Keywords:** Conservative management, False-negative biopsy, Ischemic colitis, Mass-forming, Repeat colonoscopy

## Abstract

**Rationale:**

Ischemic colitis is the most common form of intestinal ischemia, typically affecting elderly individuals with vascular risk factors such as hypertension, diabetes mellitus, atherosclerosis, and chronic kidney disease. In rare cases, a mass-forming variant may mimic malignancy or inflammatory bowel disease, creating a diagnostic challenge. We report a case of mass-forming ischemic colitis, initially suspected to be colon cancer, in which the correct diagnosis was established through short-term follow-up colonoscopy with biopsy, thereby preventing unnecessary surgical intervention.

**Patient concerns:**

A 75-year-old female presented with acute right upper quadrant abdominal pain. Her medical history included hypertension, diabetes mellitus, unstable angina, and chronic kidney disease. Abdominal CT revealed a mass-like lesion at the hepatic flexure without definitive colonic obstruction. The initial diagnosis was a suspicion of colon cancer. Colonoscopy revealed an ulcerofungating mass-like lesion with significant luminal narrowing, and the colonoscope could not pass through. However, the biopsy did not reveal any evidence of colon cancer. A follow-up colonoscopy with biopsy was performed 3 days later. The lesion showed macroscopic improvement, and the colonoscope could be advanced beyond the hepatic flexure. The biopsy result was consistent with ischemic colitis.

**Diagnoses:**

A diagnosis of mass-forming ischemic colitis was established.

**Interventions:**

She was treated conservatively with bowel rest, intravenous fluid therapy, and empirical intravenous ceftriaxone.

**Outcomes:**

Her abdominal pain gradually improved, and oral intake was resumed without complication. She was subsequently discharged in stable condition. A follow-up colonoscopy 2 months later demonstrated near-complete resolution of the previous lesion.

**Lessons:**

In the absence of colonic perforation or obstruction, short-term follow-up colonoscopy and biopsy helped distinguish mass-forming ischemic colitis from colon cancer, avoiding misdiagnosis and unnecessary surgery.

## 1. Introduction

Ischemic colitis is the most common form of intestinal ischemia, typically affecting elderly individuals with vascular risk factors such as hypertension, diabetes mellitus, atherosclerosis, and chronic kidney disease.^[1,2]^ It results from transient or persistent colonic hypoperfusion, leading to a spectrum of mucosal injury. Most cases of ischemic colitis occur in watershed areas of the colon and present with characteristic endoscopic findings such as ulceration, edema, and exudate.^[1]^ However, in rare cases, a mass-forming variant of ischemic colitis may occur, which can sometimes resemble other gastrointestinal diseases such as malignancy or inflammatory bowel disease. This mass-forming ischemic colitis is often associated with delayed or complicated diagnosis.^[3,4]^ This variant has been reported to predominantly affect elderly individuals and females and typically involves the right colon.^[5]^ Previous reports have described mass-forming ischemic colitis as a rare mimic of colorectal malignancy, with some cases requiring surgical resection for definitive diagnosis.^[4,6–8]^

However, we report a case of mass-forming ischemic colitis at the hepatic flexure that initially mimicked colon cancer, in which short-term follow-up colonoscopy and biopsy allowed us to avoid unnecessary surgery. This case report was approved by the Institutional Review Board of Jeonbuk National University Hospital (IRB No. CUH 2025-09-006). Written informed consent for publication was obtained from the patient on June 25, 2024.

## 2. Case presentation

### 2.1. Chief complaints

A 75-year-old female patient presented to the emergency department with acute right upper quadrant abdominal pain.

### 2.2. History of present illness

Prior to presentation at our hospital, the patient was admitted to a local clinic for right chest wall pain after a fall. She was diagnosed with fractures of the right 5th, 6th, and 7th ribs and was admitted for conservative management. There was no documented hypotension, dehydration, reduced oral intake, or opioid-induced constipation following the fall. During hospitalization, she developed right upper quadrant abdominal pain. The patient was referred to our institution for further evaluation.

### 2.3. History of past illness

Her medical history included hypertension, diabetes mellitus, dyslipidemia, unstable angina, and stage 3 chronic kidney disease (CKD). She also had adrenal insufficiency but was not receiving maintenance corticosteroid therapy at the time of presentation. She had no history of vasoconstrictor or diuretic use and was a never-smoker.

### 2.4. Personal and family history

No relevant family history was identified.

### 2.5. Physical examination

On physical examination, localized tenderness was noted in the right upper quadrant area.

### 2.6. Laboratory examinations

Initial laboratory investigations revealed a white blood cell count of 9.4 × 10^9^/L with neutrophil predominance (80.6%), hemoglobin of 11.8 g/dL, and a platelet count of 162 × 10^9^/L. C-reactive protein was markedly elevated at 135.2 mg/L. Serum lactate was within the normal range (1.4 mmol/L). Renal function was impaired, with a serum creatinine level of 1.44 mg/dL and an estimated glomerular filtration rate (eGFR) of 35.8 mL/min/1.73 m^2^, consistent with the patient’s known stage 3 CKD. Carcinoembryonic antigen (CEA) and carbohydrate antigen 19–9 (CA 19–9) were within the normal range (CEA < 1.8 ng/mL, CA 19–9 < 9.0 U/mL).

### 2.7. Imaging examinations

A non-enhanced abdominal computed tomography (CT) scan – performed due to the patient’s stage 3 CKD – revealed marked bowel wall thickening and pericolic fat stranding at the hepatic flexure of the colon without definitive colonic obstruction (Fig. 1). The radiologic findings raised concern for advanced colon cancer. However, because the examination was performed without intravenous contrast owing to the patient’s stage 3 CKD, the CT findings were nonspecific and could also be seen in inflammatory or ischemic colonic diseases. Therefore, correlation with the clinical presentation, endoscopic findings, and histopathologic examination was considered essential for establishing the diagnosis. After bowel preparation with a 1-L polyethylene glycol plus ascorbate solution, colonoscopy revealed an ulcerofungating mass-like lesion at the hepatic flexure with significant luminal narrowing, and the colonoscope could not pass through (Fig. 2). The differential diagnosis included colon cancer, mass-forming ischemic colitis, inflammatory bowel disease, and infectious colitis. Initial colonoscopic biopsy demonstrated nonspecific ulcerative inflammation. Although there was no histologic evidence of cancer, the possibility of malignancy remained high, and a false negative result could not be excluded. Immediate surgery was not pursued because the patient was hemodynamically stable, had no signs of complete bowel obstruction or peritonitis, and her advanced age and multiple comorbidities were associated with an increased operative risk. CT angiography was not performed because the patient’s stage 3 CKD raised concern about contrast-associated acute kidney injury, and her stable clinical condition did not indicate an urgent need for further contrast-enhanced vascular imaging. As the patient remained hemodynamically stable without ongoing abdominal pain, fever, or signs of peritonitis, an early repeat colonoscopy with biopsy was considered reasonable and was performed 3 days later to obtain a definitive diagnosis while allowing close clinical observation. On repeat colonoscopy, the lesion was still present at the hepatic flexure. However, the lesion showed macroscopic improvement compared to the initial examination, and the colonoscope was successfully advanced beyond the lesion (Fig. 3). A repeat biopsy was obtained, and the result was consistent with ischemic colitis, with no evidence of malignancy. This histopathologic finding, together with the marked macroscopic regression of the lesion on repeat colonoscopy, supported the diagnosis of mass-forming ischemic colitis and allowed colorectal malignancy to be excluded without surgical resection.

**Figure 1.**
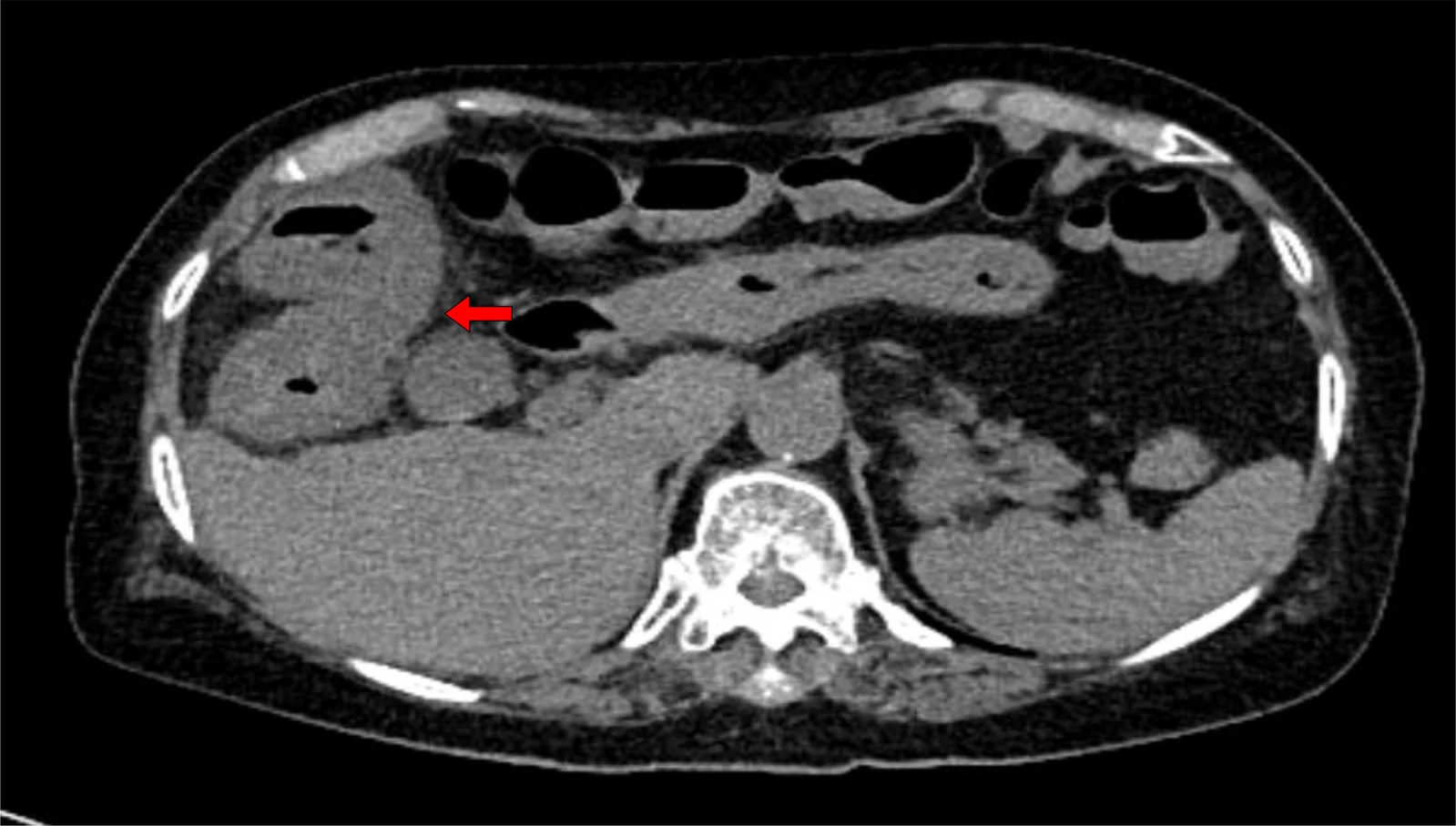
Non-enhanced abdominal computed tomography (CT) scan revealed segmental wall thickening of the colon from the distal ascending colon to the proximal transverse colon, measuring approximately 15 cm, with associated pericolic fat stranding (red arrow).

**Figure 2.**
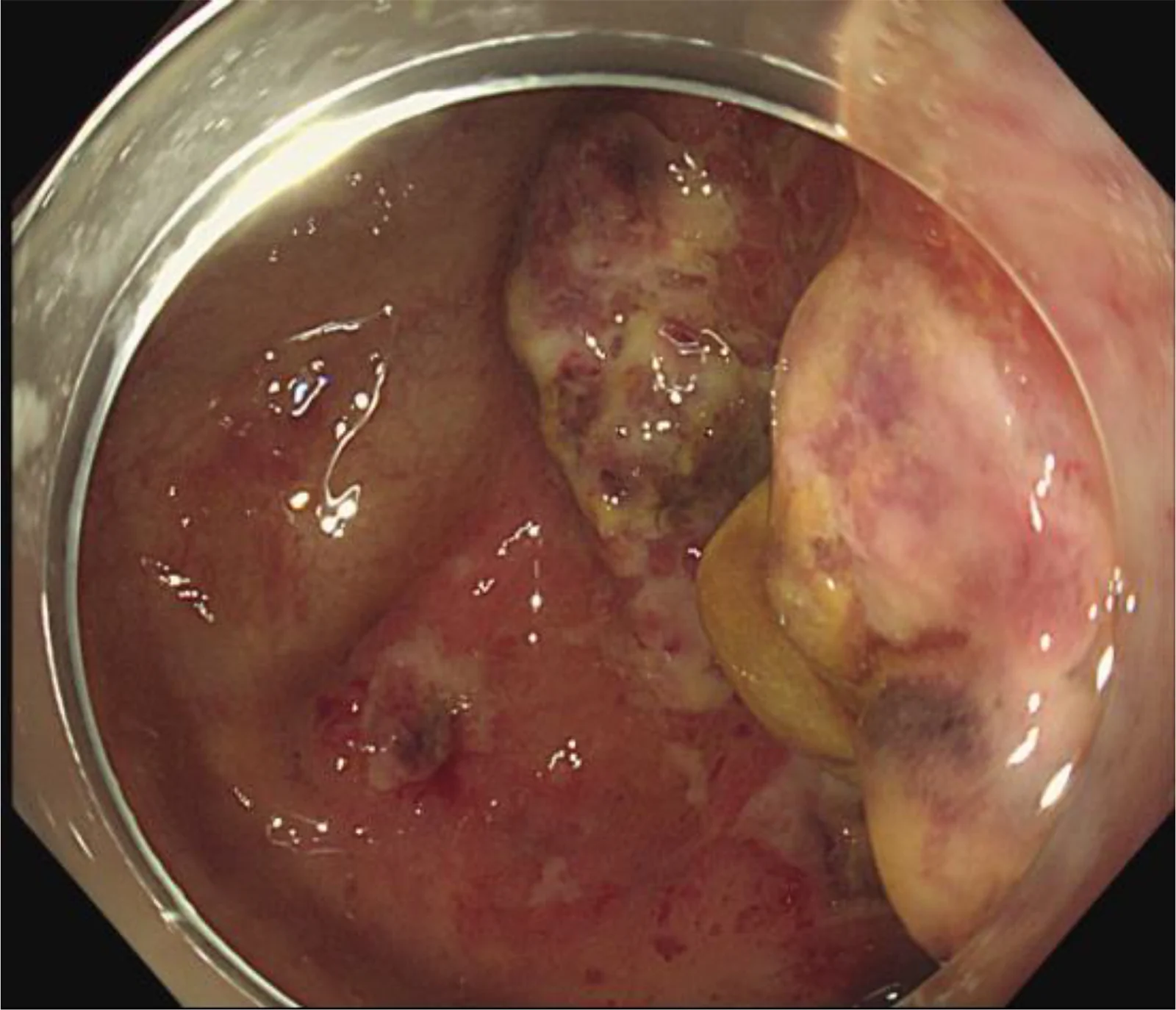
Initial colonoscopy revealed an ulcerofungating lesion at the hepatic flexure with marked luminal narrowing, preventing advancement of the colonoscope (Day 1).

**Figure 3.**
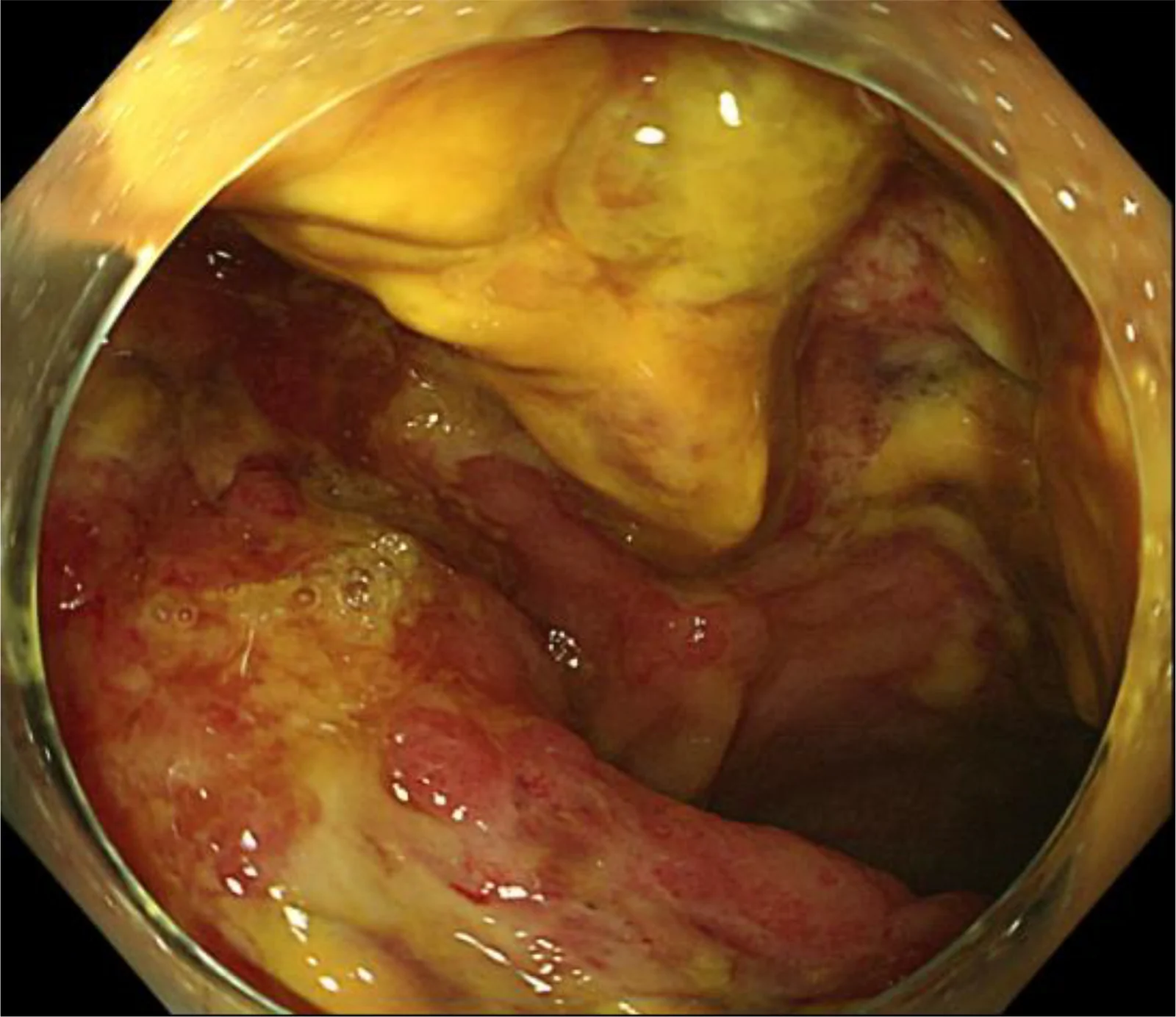
Repeat colonoscopy demonstrated the lesion at the hepatic flexure, with mild interval improvement compared to the initial examination, allowing successful advancement of the colonoscope (Day 4).

## 3. Final diagnosis

Finally, mass-forming ischemic colitis at the hepatic flexure was established.

## 4. Treatment

She was managed conservatively with bowel rest, intravenous fluid therapy, and empirical intravenous ceftriaxone because infectious colitis could not initially be excluded. Blood cultures showed no growth, and stool cultures were not performed. Hemodynamic status was monitored with regular vital sign assessments throughout hospitalization. No anticoagulant or antiplatelet therapy was administered during hospitalization because of the concern for gastrointestinal bleeding. After the biopsy results from the second colonoscopy became available, oral intake was resumed with sips of water because there was no worsening of abdominal pain or gastrointestinal bleeding, and the diet was subsequently advanced to a soft diet as tolerated. Her abdominal pain gradually improved, and she was discharged in stable condition after an 11-day hospitalization. The patient expressed relief that surgical intervention could be avoided and that her symptoms improved with conservative treatment.

## 5. Outcome and follow-up

She was followed up in the outpatient clinic, and a follow-up colonoscopy performed approximately 2 months later demonstrated marked mucosal healing, with near-complete resolution of the lesion at the hepatic flexure (Fig. 4). The overall clinical course is summarized in Table 1.

**Table 1:** Timeline of the clinical course.

Time	Clinical course
Day 0	Acute right upper quadrant pain. Non-enhanced abdominal CT demonstrated a mass-like lesion at the hepatic flexure, suspicious for colon cancer.
Day 1	Colonoscopy revealed an ulcerofungating lesion with severe luminal narrowing. Initial biopsy showed nonspecific ulcerative inflammation without evidence of malignancy.
Day 4	Repeat colonoscopy demonstrated macroscopic regression of the lesion. Repeat biopsy result was consistent with ischemic colitis.
Day 5–10	Conservative treatment with bowel rest and intravenous fluids. Symptoms gradually improved and oral intake was resumed.
Day 11	Discharged in stable condition without surgical intervention
2-month follow-up	Follow-up colonoscopy demonstrated near-complete resolution of the lesion.

**Figure 4.**
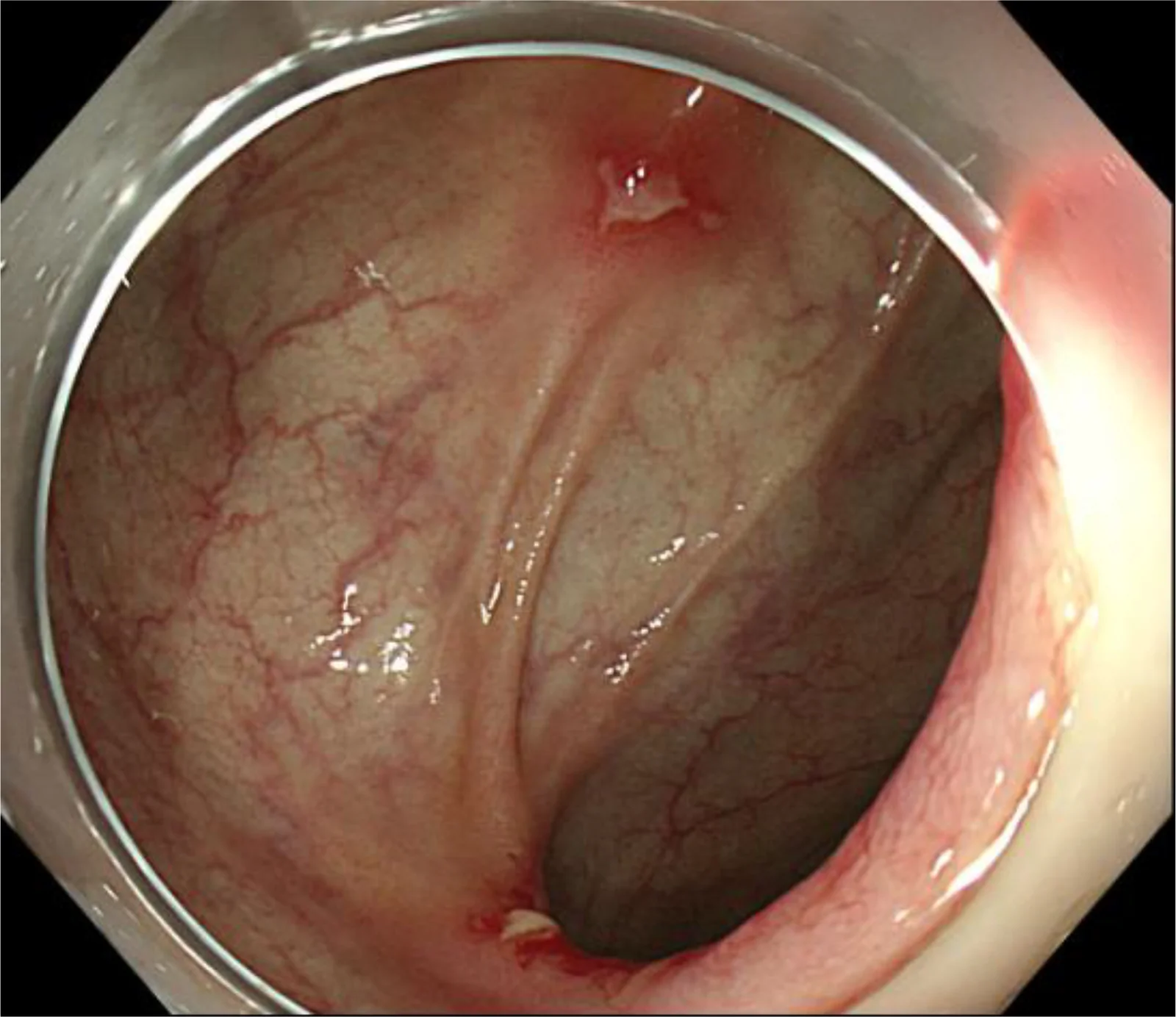
Follow-up colonoscopy demonstrated marked mucosal healing with near-complete resolution of the lesion at the hepatic flexure (2 months).

## 6. Discussion

Ischemic colitis is the most common form of gastrointestinal ischemia, with an incidence that has increased from approximately 6.1 to 22.9 cases per 100,000 person-years over recent decades, likely due to increased life expectancy and the rising prevalence of vascular comorbidities.^[3]^ It is broadly classified into occlusive and nonocclusive types. nonocclusive ischemia, the most common form, typically results from transient hypoperfusion due to hypotension, heart failure, or postoperative low-flow states.^[1,2]^ Occlusive ischemia is less frequent and involves vascular obstruction, such as arterial thrombosis, embolism, or mechanical causes like volvulus or tumor.^[1,2,9]^ Both types commonly affect the splenic flexure and rectosigmoid junction – so-called “watershed areas” of the colon that are particularly vulnerable due to relatively poor collateral circulation.^[1,9]^ In contrast, ischemic colitis can also involve the right colon – including the cecum, ascending colon, and hepatic flexure. It accounts for only about 10% of all cases, with hepatic flexure involvement being particularly uncommon.^[5,9]^

The etiology of ischemic colitis is diverse; however, diagnosis is primarily based on a combination of clinical presentation, radiologic imaging, and colonoscopic findings, along with histopathologic confirmation.^[1,2,10]^ Typically, the colonoscopic findings of ischemic colitis include segmental involvement with pale or erythematous mucosa, loss of normal vascular pattern, petechiae, friability, and superficial linear or serpiginous ulcerations. In more severe cases, circumferential ulceration or stricture formation may occur.^[3,10]^ These findings generally help differentiate ischemic colitis from malignancy or inflammatory bowel disease.^[5]^

However, ischemic colitis can rarely present in a mass-forming pattern. The presence of a mass-forming ulcerofungating lesion with luminal narrowing raised strong suspicion of advanced colon cancer, and it may pose a diagnostic difficulty for clinicians. Although CT plays an important role in the evaluation and preoperative localization of colorectal cancer, imaging findings may not be specific for malignancy and should be interpreted in conjunction with clinical, endoscopic, and histopathologic findings.^[11]^ Mass-forming ischemic colitis is a rare subtype of ischemic colitis that is frequently misinterpreted as colon cancer. This mass-forming ischemic colitis has been reported to predominantly affect elderly individuals, females, and typically involves the right colon.^[5,12]^ Our patient also had multiple vascular risk factors, including hypertension, diabetes mellitus, and angina, and was an an elderly female. Previous reports have emphasized the difficulty of distinguishing mass-forming ischemic colitis from colorectal cancer, with some cases requiring surgical resection to establish the final diagnosis.^[4,6–8]^ In our case, however, the combination of an early repeat colonoscopy at 3 days, demonstrable macroscopic regression of the lesion, and repeat biopsy findings supported a diagnosis of ischemic colitis and allowed surgery to be avoided. This approach may be particularly valuable in elderly patients with substantial comorbidity, in whom unnecessary colectomy carries considerable procedural risk.

Although the initial biopsy showed no evidence of malignancy, colorectal cancer remained a major diagnostic consideration because severe luminal stenosis limited complete endoscopic evaluation, and extensive ulceration and necrotic tissue may have resulted in sampling error. The decision to perform an early repeat colonoscopy was based on the patient’s stable clinical condition, with no ongoing abdominal pain, fever, or signs of peritonitis. In the absence of clinical evidence of perforation or complete colonic obstruction, repeat colonoscopy was considered reasonable, despite the potential risk of perforation associated with endoscopic examination of an ischemically injured colon. Repeat colonoscopy with careful endoscopic manipulation was performed 3 days later to reassess the lesion after a short period of conservative management, allowing repeat histologic assessment while avoiding unnecessary delay in excluding colorectal malignancy.

Therefore, a repeat colonoscopy was performed to obtain further histologic evidence and reassess the lesion. Serial colonoscopic examinations demonstrated gradual improvement of the lesion, and the second biopsy also showed no evidence of cancer, making ischemic colitis the more likely diagnosis, which justified a conservative, nonsurgical approach. The patient’s right upper quadrant pain gradually resolved following conservative treatment, and follow-up colonoscopy demonstrated near-complete mucosal healing, reinforcing the role of observation and repeat endoscopy in selected cases of suspected ischemic colitis.^[3,8,13]^

To explain the tumor-like colonic lesions observed in this case, it is essential to consider the underlying pathophysiology of mass-forming ischemic colitis. The unusual tumor-like presentation of mass-forming ischemic colitis is thought to arise from severe or prolonged hypoperfusion, leading to extensive submucosal and mural changes, including edema, fibrosis, and occasionally cholesterol embolization.^[5–7]^ These morphologic alterations may account for the exophytic or stricture-like configuration observed on endoscopic or radiologic assessment. Unlike typical cases of ischemic colitis, which are usually confined to mucosal injury, this form can appear infiltrative and neoplastic, thereby mimicking carcinoma. Recognizing this pathophysiologic basis is critical in guiding appropriate management decisions and avoiding unnecessary surgical intervention, especially in patients who are clinically stable and able to tolerate a period of close observation. As endoluminal and organ-preserving technologies continue to evolve, accurate diagnosis of colorectal lesions may become increasingly important for avoiding unnecessary surgical resection and selecting the least invasive, appropriate treatment strategy.^[14]^

This case report has several limitations. First, as a single case report, the findings may not be generalizable to all patients with mass-forming ischemic colitis. Second, detailed histopathologic descriptors and microscopic images were unavailable for retrospective review, limiting further histopathologic characterization of the lesion. Third, although empirical intravenous ceftriaxone was administered because infectious colitis could not initially be excluded, no causative organisms were identified in blood cultures, and stool cultures were not performed. Therefore, an infectious etiology could not be definitively excluded microbiologically. Finally, the patient was lost to follow-up after the 2-month colonoscopy, precluding assessment of her longer-term clinical course and limiting our ability to confirm durable exclusion of colorectal malignancy.

## 7. Conclusion

This case emphasizes the importance of considering mass-forming ischemic colitis in elderly patients with multiple vascular risk factors who present with lesions suspicious for colon cancer. In the absence of colonic perforation or obstruction, short-term follow-up colonoscopy with biopsy may help avoid misdiagnosis and unnecessary surgery.

## Author contributions

**Writing – original draft:** Hyeon Seok Baek.

**Writing – review & editing:** Seung Young Seo.
